# Mitigating cathode biofouling in membrane-less MFCs using CuO-doped activated carbon: a comparative study of batch and continuous modes

**DOI:** 10.1038/s41598-025-27118-2

**Published:** 2025-11-23

**Authors:** Nasser A. M. Barakat, Hazem Gamal, Rania Osama

**Affiliations:** 1 Chemical Engineering Department, Faculty of Engineering, Minia University, El-Minia, 61516 Egypt; 2 Civil Engineering Department, Faculty of Engineering, Minia University, El-Minia, 61516 Egypt

**Keywords:** Microbial fuel cell, CuO-doped activated carbon, Antibacterial cathode, Continuous operation, Wastewater treatment, Biotechnology, Engineering, Environmental sciences, Materials science, Microbiology

## Abstract

A membrane-less microbial fuel cell system was developed and evaluated using CuO-incorporated activated carbon (CuO/AC) cathodes designed for dual functionality: enhanced oxygen reduction reaction activity and antibacterial performance. CuO/AC composites were synthesized via wet impregnation followed by thermal treatment, and the 10wt% CuO/AC formulation demonstrated the best performance. The optimized cathode achieved a maximum power density of ~ 1.25 W/m^2^ and a peak current density of ~ 5.2 A/m^2^, significantly outperforming pristine AC and AC-CNTs cathodes. Long-term stability tests showed that the 10wt% CuO/AC cathode maintained an open cell potential around 0.85–1.0 V in batch mode for over 40 days, while AC-CNTs cathodes exhibited lower open cell potential and severe performance degradation due to biofouling. Microscopic analysis confirmed heavy biofilm and microbial colony formation on AC-CNTs cathode, whereas the CuO-containing cathode surface remained clean and free from microbial colonization. Moreover, MFCs operated in continuous mode demonstrated superior operational stability and higher COD removal efficiency (~ 85.3%) compared to batch mode (~ 76.0%), despite slightly lower OCP and initial power densities. These findings highlight the synergistic role of CuO in enhancing cathodic performance and biofouling resistance, while also demonstrating the industrial relevance of continuous mode operation for stable and efficient wastewater treatment coupled with energy recovery.

## Introduction

The rapid industrial expansion in recent decades has led to the significant discharge of wastewater streams laden with organic pollutants, posing serious environmental challenges. Among these, industrial effluents from the food and sugar industries are particularly problematic due to their high COD and biological oxygen demand BOD, which severely threaten aquatic ecosystems and public health if left untreated^1–3^.

Traditional treatment methods such as activated sludge, anaerobic digestion, and chemical oxidation are widely used for treating industrial wastewater^4,5^. However, these approaches are generally energy-intensive and economically unsustainable in the long term. Consequently, there is an urgent need to develop alternative technologies that not only reduce pollutants effectively but also offer additional value—particularly in the form of energy recovery^6^.

One such promising alternative is the microbial fuel cell (MFC)—a bioelectrochemical system capable of degrading organic matter while simultaneously generating electrical energy. In contrast to conventional wastewater treatment methods, MFCs convert chemical energy from biodegradable compounds into electricity via the metabolic activity of electroactive microorganisms. Thus, MFCs represent an environmentally friendly, energy-positive solution to the problem of organic wastewater treatment^7–11^.

A typical MFC comprises two electrodes—an anode and a cathode—separated by a membrane. Electroactive bacteria at the anode oxidize organic matter, releasing electrons that flow through an external circuit to the cathode, where they reduce an electron acceptor (typically oxygen). In recent years, air-cathode single-chamber MFCs have gained attention due to their ability to utilize atmospheric oxygen directly as the terminal electron acceptor, eliminating the need for expensive and hazardous chemical oxidants used in traditional two-chamber designs. This simplification reduces cost and enhances scalability^12^.

Membranes, commonly employed in MFCs to prevent oxygen diffusion to the anode and separate microbial communities, also help in retaining protons and improving selectivity. However, their presence significantly increases the internal resistance of the cell, thereby diminishing the power output. Moreover, membranes are often costly and prone to fouling, which further limits their long-term use^13,14^.

To address this issue, membrane-less MFCs have been proposed^15^. While they simplify the design and reduce cost, they introduce a new challenge: biofouling of the cathode surface. In the absence of a physical barrier, aerobic microorganisms tend to colonize the cathode, forming a biofilm that disrupts oxygen reduction and leads to substantial power loss due to circuit disconnection^16,17^.

A promising strategy to combat this limitation involves the incorporation of antibacterial agents into the cathode material. Among various antimicrobial additives such as silver nanoparticles, TiO_2_, and ZnO, CuO stands out due to its strong antibacterial properties, low cost, and favorable oxygen reduction reaction (ORR) activity^18,19^. Additionally, CuO’s stability in aqueous environments makes it an excellent candidate for long-term MFC operation. To further enhance the cathode’s performance, CNTs can be added in small quantities to boost the electrical conductivity and surface area, thereby improving ORR kinetics^20,21^.

From an industrial standpoint, operating microbial fuel cells in continuous mode offers several practical and economic advantages over traditional batch systems. Industrial wastewater treatment processes typically run continuously, with effluents generated around the clock^22^. Therefore, integrating a batch-mode MFC would require significant storage infrastructure and complex scheduling to synchronize treatment cycles, which is inefficient and impractical on a large scale^23^. In contrast, continuous mode operation aligns seamlessly with real-world industrial discharge patterns, enabling real-time treatment and steady-state energy production. This operational mode not only ensures a consistent processing flow but also facilitates scalability and automation, which are critical for full-scale implementation^24^. In terms of performance, transitioning to continuous mode can significantly influence both the anodic and cathodic reactions within the MFC. On the anode side, the continuous influx of substrate-rich wastewater ensures a more uniform supply of nutrients to the electroactive biofilm, potentially enhancing microbial metabolism and electron generation. Moreover, the constant movement of the fluid minimizes the accumulation of inhibitory metabolic byproducts near the anode, which are known to suppress bacterial activity in static systems. On the cathode side, fluid motion improves mass transfer dynamics, especially the availability of dissolved oxygen for the ORR^25^. This can result in a higher and more stable cathodic potential. Importantly, the continuous flow also helps reduce the thickness and compactness of unwanted biofilms that form on the cathode in membrane-less configurations, as the shear force generated by the moving liquid can physically dislodge or suppress microbial colonization. This mitigates the disconnection issues commonly observed in fouled cathodes, maintaining ORR efficiency over longer periods. Overall, continuous mode operation can lead to enhanced power output, improved operational stability, and extended electrode lifespan, making it a more viable solution for long-term industrial applications^26^. Despite the growing interest in continuous operation for practical applications, few studies have reported on continuous mode MFCs, especially those utilizing membrane-less designs. This study aims to fill this gap by investigating the influence of flow mode on the performance, stability, and longevity of the system.

In this study, we designed and evaluated a CuO-doped AC cathode integrated with CNTs, serving a dual function: suppressing microbial colonization and promoting efficient ORR. The CuO/AC-CNT cathode was tested in a membrane-less MFC fueled by real industrial wastewater from Abu-Korkas Sugar Factory without the addition of external microbial inoculum—relying entirely on indigenous bacteria. Furthermore, a large-scale MFC was assembled with eight graphitized corncob anodes and eight CuO/AC-CNT cathodes. The system was operated for 40 days under both batch mode and continuous flow mode using a circulation rate of 1.9 L/h, equivalent to a linear velocity of 3.4 m/min.

## Experimental section

### Materials

Commercial AC was purchased from Merck (Germany). Copper(II) nitrate trihydrate (Cu(NO_3_)_2_·3H_2_O), ethanol, polyvinylidene fluoride (PVDF), and N, N-dimethylformamide (DMF) were all analytical grade and used as received. Multi-walled CNTs were procured from Sigma-Aldrich. Carbon cloth (CC) sheets were used as the substrate for cathode preparation. Real industrial wastewater was obtained from Abu-Korkas Sugar Factory (Egypt) and used without any external inoculation. All other chemicals were of analytical grade and used without further purification.

### Synthesis of CuO-incorporated activated carbon

CuO-doped activated carbon was synthesized using a modified wet impregnation method. Briefly, specified weights of copper nitrate (0, 1, 3, 10, 15 and 10 wt% relative to AC) were dissolved in 5 mL of ethanol under constant stirring. Then, 1 g of activated carbon was added to the solution to form a slurry. The mixture was heated to evaporate ethanol while maintaining uniform mixing. The resulting semi-dried material was then oven-dried at 110 °C for 5 h, followed by calcination at 280 °C for 3 h in air. This process resulted in uniform distribution of CuO particles over the AC surface, yielding a composite material with antibacterial and ORR activity.

### MFC electrodes preparation

#### Anode preparation

The anode was fabricated using corncob biomass as a sustainable and low-cost carbon precursor as it was explained in our previous study^17^. Initially, the raw corncobs were thoroughly washed with tap water to remove surface dust and impurities, followed by rinsing several times with deionized water to eliminate any remaining contaminants. The cleaned corncobs were then dried in an oven at 80 °C for 12 h to ensure complete moisture removal. After drying, a high-corrosion-resistant stainless-steel rod (3 mm diameter) was carefully inserted along the axial center of each corncob piece to act as a current collector during MFC operation.

Subsequently, the assembled corncob rods were placed in a closed-atmosphere quartz tube furnace and subjected to graphitization at 1000 °C for 2 h under a continuous removing the exhaust gases as bubbles in water beaker to prevent air entering. This thermal treatment converted the organic structure into a conductive graphitized carbon material, enhancing its electrical conductivity, surface area, and mechanical strength. The resulting graphitized corncob electrodes were used directly as anodes in the MFC system without further modification. This configuration provided a robust, porous, and biocompatible surface for microbial colonization and efficient electron transfer.

####  Cathode preparation

To fabricate the cathode, 1 g of PVDF was dissolved in 8 mL of DMF to create a uniform binder solution. To this, 0.5 g of CuO/AC powder was added, and the mixture was stirred thoroughly. Subsequently, 0.035 g of CNTs was incorporated into the slurry and further stirred until a homogeneous ink was obtained. One face of a carbon cloth sheet was then coated with this ink using a brush-coating technique. The coated cathodes were dried at 60 °C for 2 h and then thermally treated at 300 °C to enhance structural integrity and catalytic properties.

### MFC design and assembly

A large-size single-chamber, membrane-less microbial fuel cell was assembled, consisting of eight anodes and eight cathodes, arranged to maximize surface exposure and interaction. The anodes and cathodes were fixed on opposing sides of an acrylic chamber using non-conductive holders. The inter-electrode distance was approximately 4 cm. Each anode-cathode pair was connected externally via titanium wire and operated in open circuit mode for most of the experiment.

**Fig. 1:**
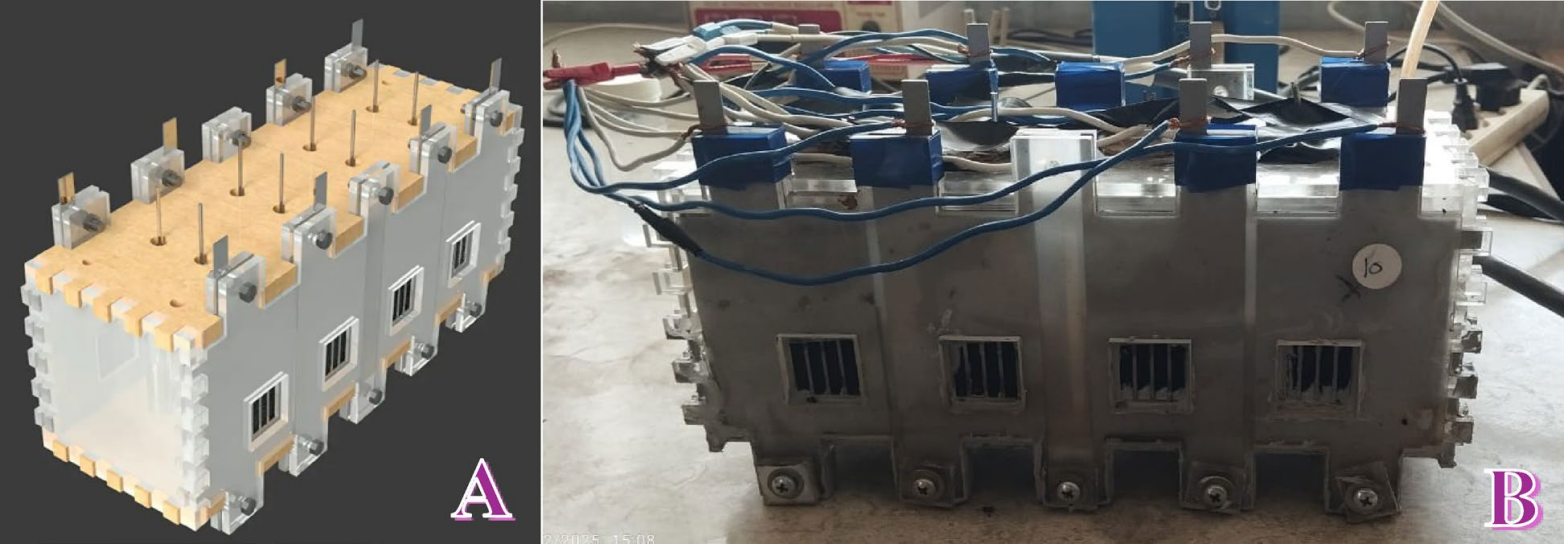
(**A**) Schematic diagram and (**B**) photo image for the designed microbial fuel cell.

The MFC was fueled with real industrial wastewater from Abu-Korkas Sugar Factory, used directly without any pretreatment or external microbial seeding. The system was run under two operational modes: (i) Batch mode, where the wastewater was kept stagnant for a fixed period, and (ii) Continuous mode, where the wastewater was circulated using a peristaltic pump at a flow rate of 1.9 L/h, corresponding to a fluid linear velocity of approximately 3.4 m/min within the chamber. The membrane-less MFC system used in this study consisted of a custom-fabricated acrylic chamber housing eight anodes and eight cathodes. Figure 1 shows a schematic diagram and a photo image for the used cell. The anodes, prepared from graphitized corncob rods, each embedded with a high corrosion-resistant stainless-steel wire (2 mm diameter), were inserted axially to serve as a current collector. These anodes were vertically arranged into two parallel columns, each consisting of four anodes, and fully immersed in the industrial wastewater solution.

Each side wall of the acrylic chamber was designed with four square openings, into which the air cathodes were mounted. Passive oxygen diffusion from air into the cathode surface occurred naturally without forced aeration. These cathodes, composed of catalyst-coated carbon cloth with a stainless-steel sheet as current collector, were securely sealed using silicone rubber and tightly fixed to the chamber using Teflon tape and ten stainless-steel screw pins. This sealing strategy effectively prevented electrolyte leakage while maintaining exposure of the cathode’s outer surface to atmospheric oxygen, enabling air-cathode functionality. A schematic diagram and a photograph of the assembled MFC setup are provided to visually support the described configuration (see Fig. 1).

The working volume of the assembled MFC was approximately 2.5 L. The anodes were cylindrical graphitized corncobs (~ 10 cm in length, ~ 2.5 cm in diameter), resulting in an apparent surface area of ~ 78.5 cm^2^ per anode. The cathodes were prepared by coating one face of a 5 cm × 5 cm carbon cloth sheet (total area = 25 cm^2^), but the active area used for electrochemical reaction was restricted by the current collector design. Specifically, each stainless-steel sheet current collector featured four vertical rectangular openings (5 mm × 26 mm each), giving an exposed area of 5.2 cm^2^ per cathode. This area was considered the effective surface area for oxygen reduction and was used to normalize both power and current densities. Current and power outputs were calculated based on Ohm’s law using the following equations, and the values were normalized to the apparent (geometrical) surface area of the exposed face of the carbon cloth cathode (8 × 0.52 cm^2^). This normalization approach was selected to ensure consistency in comparing different cathode configurations.

1 $$\:P.D=\frac{V\times\:I}{A}$$

2 $$\:\:\:\:\:\:\:\:\:C.D=\frac{I}{A}$$

where P.D is the power density (W/m^2^), C.D is the current density (A/m^2^), *V* and *I* are the measured voltage (Volt) and current (Ampere), respectively, and *A* is the total active cathode surface area. The electrochemical performance of the fabricated MFCs was assessed using linear sweep voltammetry (LSV) with a VersaSTAT 4.0 Potentiostat. The polarization curves (voltage vs. current) were obtained by sweeping the cell voltage while recording the output current.

During the experimental period, the MFCs were primarily operated under closed-circuit conditions, with external resistors connected to enable electron flow between the anode and cathode. Open circuit potential (OCP) measurements were conducted periodically for short durations to assess the electrochemical behavior of the cells but were not used as the default operational state. This approach ensured that the system functioned as a microbial fuel cell rather than shifting toward anaerobic digestion processes typically associated with open-circuit or idle conditions.

The characteristics of the used industrial wastewater are summarized in Table 1.

**Table 1:** Characteristics of the used industrial wastewaters.

pH	TSS	TDS	COD	BOD
**5 ~ 6.9**	**100 ~ 150**	**2000 ~ 2500**	**2000 ~ 2200**	**1500 ~ 1700**

The electrical performance of the MFC was monitored over a 40-day operation period. Electrochemical measurements were conducted using a potentiostat, with the anode connected to the working electrode, and the cathode connected to both the counter and reference electrodes in a standard three-electrode configuration. A GL220 midi-logger was employed to continuously record the output voltage during operation. Power and current densities were calculated and normalized to the projected surface area of the cathode^27,28^.

### Antibacterial test

To evaluate the antibacterial properties of the synthesized CuO-incorporated activated carbon, a microbial colony counting assay was conducted using real wastewater as a source of aerobic microorganisms. The experimental protocol involves a spread plate technique on nutrient agar dishes, which is a widely accepted method for assessing the antibacterial activity of materials against environmental bacterial populations. A fixed volume of wastewater rich in microbial content is mixed with varying concentrations of the test material (in this case, CuO/AC at 1, 2, 3, and 4 wt%. After brief incubation to allow interaction between the bacteria and the CuO/AC powder, aliquots of the treated solutions are spread evenly over nutrient agar plates. The plates are incubated at 37 °C for 24–48 h, and the number of resulting colony-forming units (CFUs) is counted as an indicator of surviving viable bacteria. A control sample without CuO/AC (0%) is used to establish the baseline microbial population.

### Characterization

The structural and morphological properties of the synthesized CuO/AC materials were examined using X-ray diffraction (XRD) and scanning electron microscopy (SEM). XRD was performed using a Bruker D8 Advance diffractometer with Cu Kα radiation to confirm the presence and phase purity of CuO nanoparticles. SEM imaging and elemental mapping (EDX) were carried out using a JEOL JSM-6510LV microscope to investigate the surface morphology and distribution of copper on the AC surface. These analyses confirmed the successful decoration of CuO and uniform distribution across the carbon surface.

## Results and discussion

### Characterization of CuO/AC electrocatalyst

**Fig. 2:**
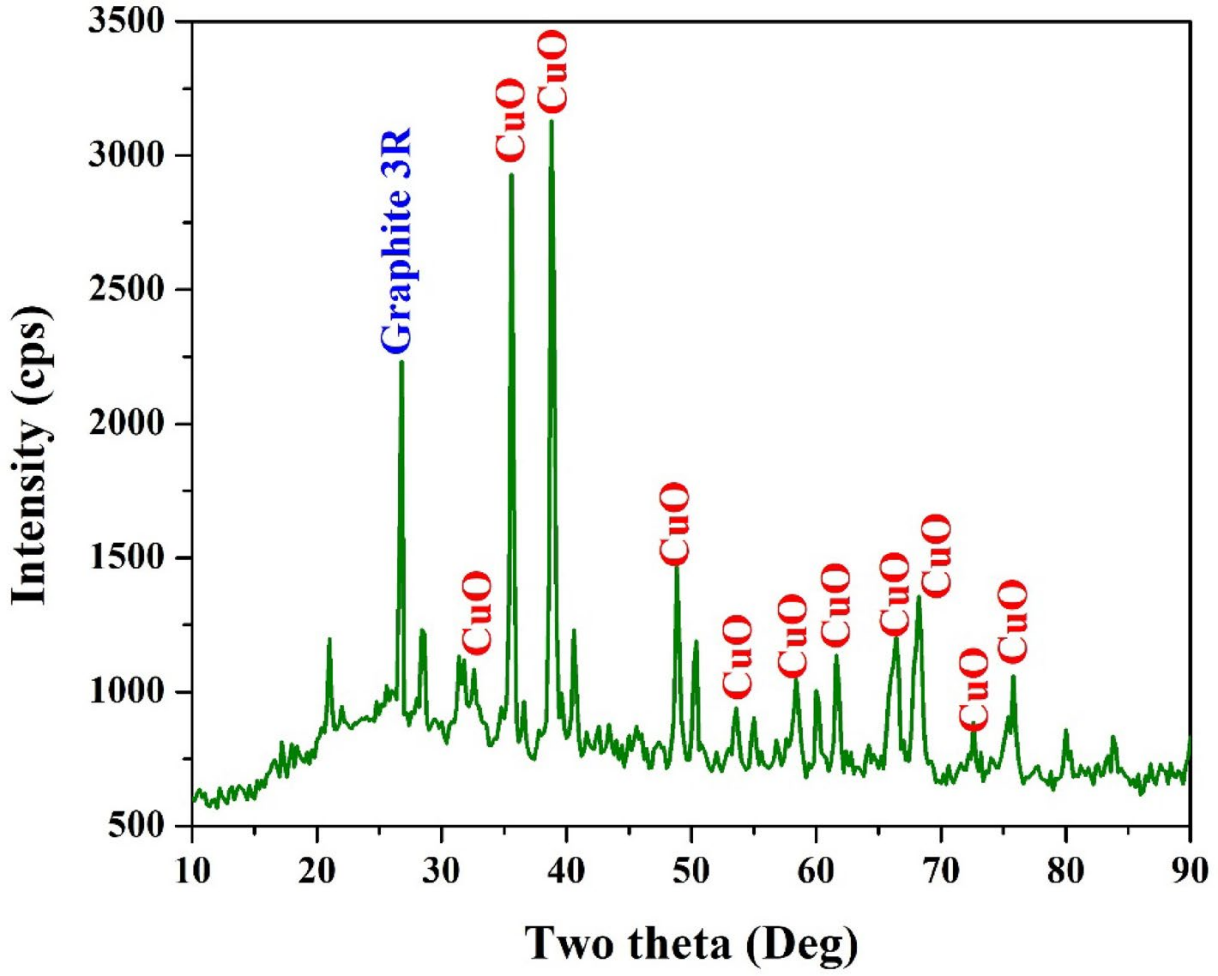
XRD pattern for the treated activated carbon using 10 wt% Cu(NO_3_)_2_ solution.

The crystalline structure of the synthesized CuO/AC was investigated using XRD, and the resulting diffraction pattern for the 10 wt% Cu(NO_3_)_2_ sample is shown in Fig. 2. The XRD profile clearly reveals the successful formation of crystalline CuO nanoparticles along with graphitized carbon phases.

The diffraction peaks corresponding to CuO are observed at 2θ = 32.5°, 35.2°, 35.5^o^, 38.7°, 38.9^o^, 48.8°, 58.3°, 61.5°, 65.2° and 66.3°, which match well with the standard monoclinic CuO phase (ICDD PDF Card No. 01–048−1548). These peaks correspond to the crystallographic planes (110), (002), (11 − 1), (111), (220), (20 − 2), (202), (11 − 3), (022) and (31 − 1) respectively. The presence of sharp and well-defined CuO peaks confirms the successful decomposition of copper nitrate into CuO upon thermal treatment at 280 °C. The mechanism likely involves the following sequential steps: Thermal decomposition of Cu(NO_3_)_2_ during heating in air initiates at relatively low temperatures^29^:

3 $$\begin{aligned}{\bf{Cu}}{\left( {{\bf{N}}{{\bf{O}}_{\bf{3}}}} \right)_{\bf{2}}} \cdot {\bf{3}}{{\bf{H}}_{\bf{2}}}{\bf{O}}~ \to {\text{ }}{\bf{Cu}}{\left( {{\bf{N}}{{\bf{O}}_{\bf{3}}}} \right)_{\bf{2}}} + {\text{ }}{\bf{3}}{{\bf{H}}_{\bf{2}}}{\bf{O}} \uparrow\end{aligned}$$

 

Continued heating leads to the decomposition of the anhydrous nitrate:

4 $$\begin{aligned} {\bf{2Cu}}{\left( {{\bf{N}}{{\bf{O}}_{\bf{3}}}} \right)_{\bf{2}}} \to {\bf{2CuO}}{\text{ }} + {\text{ }}{\bf{4N}}{{\bf{O}}_{\bf{2}}} \uparrow {\text{ }} + {\text{ }}{{\bf{O}}_{\bf{2}}} \uparrow\end{aligned}$$

This reaction occurs in the presence of activated carbon, which may help anchor the forming CuO nanoparticles due to the high surface area and porous nature of the AC support. In addition to CuO, a graphitic carbon peak at 2θ ≈ 26.5° is observed and indexed as Graphite-3R, corresponding to the (002) plane of rhombohedral graphite structure (ICDD PDF Card No. 01–026−1079). The presence of this phase indicates partial graphitization of the AC, possibly enhanced by the thermal conditions during the drying and calcination processes, or may result from graphitized impurities present in the commercial AC.

The coexistence of CuO and graphitized carbon in the XRD pattern Indicates a successful synthesis of a composite catalyst, where CuO serves as the electrocatalytically active component and the conductive carbon matrix enhances electron transfer. The distinct peak intensities and absence of residual nitrate peaks further support the complete decomposition of the precursor and the formation of phase-pure CuO crystals.

**Fig. 3:**
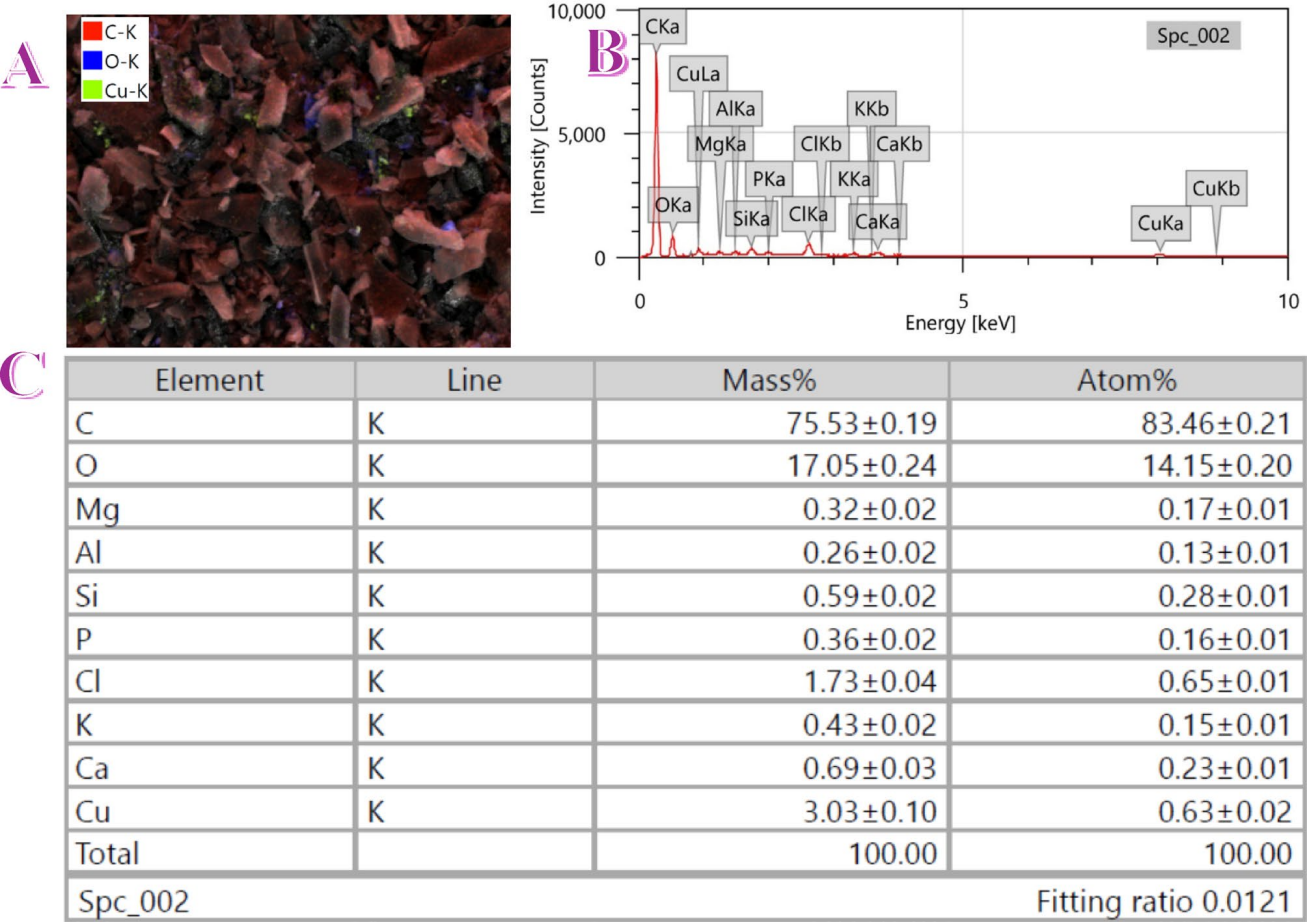
(**A**) SEM image of the CuO-doped activated carbon sample containing 10 wt% Cu(NO_3_)_2_, captured at ×15,000 magnification, (**B**) EDX spectrum and (**C**) quantitative elemental analysis for the produced sample.

The morphology and elemental composition of the CuO/AC sample containing 10 wt% copper nitrate were analyzed using SEM combined with energy-dispersive X-ray spectroscopy (EDX). The results are presented in Fig. 3, which includes three panels: the SEM micrograph (upper left), EDX spectrum (upper right), and the quantitative EDX elemental table (lower panel).

Figure 3A presents the elemental mapping image of the CuO-incorporated activated carbon cathode, offering a clearer visualization of the surface distribution of key elements. The image uses color-coded overlays—red for carbon (C-K), blue for oxygen (O-K), and green for copper (Cu-K)—to represent the elemental composition. As seen, the carbon signal dominates the background matrix, forming the structural backbone of the cathode. Scattered blue signals confirm the uniform presence of oxygen, while distinct green spots, observed across the image, confirm the localized deposition of copper species.

The co-localization of oxygen and copper strongly supports the presence of CuO particles. These particles appear embedded within the porous carbon structure, providing direct evidence of the angular, crystalline-like CuO domains described in the XRD and SEM analyses. Importantly, the green regions are not agglomerated but rather finely distributed, which is critical for maximizing electrochemical accessibility and ensuring consistent catalytic activity across the electrode surface. This confirms the successful formation and stable dispersion of CuO on the AC matrix—a key factor contributing to the improved performance observed in the microbial fuel cell.

The corresponding EDX spectrum **(**Fig. 3B**)** confirms the presence of copper, as evidenced by the prominent Cu Kα and Cu Lα peaks at approximately 8.0 keV and 0.9 keV, respectively. The quantitative EDX data **(**Fig. 3C**)** show that Cu is present at a substantial concentration—3.03 wt% (equivalent to 0.63 atom%)—verifying the successful incorporation of CuO onto the carbon support. This concentration aligns well with the intended CuO loading derived from the 10 wt% Cu(NO_3_)_2_ precursor, supporting the efficiency of the impregnation and calcination synthesis method.

In addition to Cu, the EDX analysis detected a high content of carbon (75.53 wt%) and oxygen (17.05 wt%), which correspond to the AC backbone and the oxygen-containing functional groups or metal oxides, respectively. The relatively high oxygen content further supports the formation of CuO rather than metallic copper or Cu₂O. Minor amounts of other elements such as Mg, Al, Si, P, Cl, K, and Ca were also detected. These elements are likely trace impurities originating from the raw AC material or residues from the synthesis process. Notably, Cl and K may originate from the precursor purification or residual salts.

**Fig. 4:**
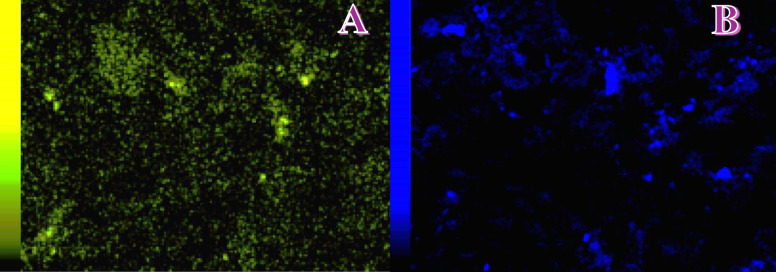
Copper; (**A**) and oxygen; (**B**) elemental distribution fir the CuO/AC (10 wt% sample),.

To further validate the elemental composition and spatial distribution of the active components in the CuO/AC composite, elemental mapping analysis was conducted. The panel A of Fig. 4 displays the distribution of copper, while the panel B shows the distribution of oxygen across the surface of the 10 wt% CuO/AC sample.

The copper elemental map (yellow-green intensity scale) exhibits a well-dispersed pattern, indicating that copper species are homogeneously distributed over the activated carbon surface. The widespread and relatively uniform signal intensity confirms the effective impregnation and anchoring of CuO nanoparticles onto the porous carbon substrate. A few brighter regions indicate minor local agglomerations, but these are not dominant and are likely due to natural variations in pore structure or surface chemistry of the AC.

The oxygen elemental map (blue intensity scale) complements the Cu distribution and shows a similarly widespread presence of oxygen atoms, consistent with the formation of CuO and the intrinsic oxygen-containing functional groups in the activated carbon matrix. The overlapping presence of both Cu and O throughout the matrix supports the conclusion that the observed copper phase is indeed in the oxidized form, rather than metallic Cu or Cu_2_O, as also confirmed by XRD results.

This uniform distribution of both Cu and O elements is critical for maximizing the electrochemically active surface area and ensuring the consistency of the cathode performance across its surface. Furthermore, the even dispersion of CuO particles is expected to provide uniform antibacterial activity, minimizing localized fouling and improving long-term electrode stability in membrane-less microbial fuel cell operation. Overall, the elemental mapping images provide strong evidence that the synthesis method yielded a homogeneously distributed CuO coating over the carbon support, reinforcing the success of the material design for dual-function MFC cathode applications.

**Fig. 5:**
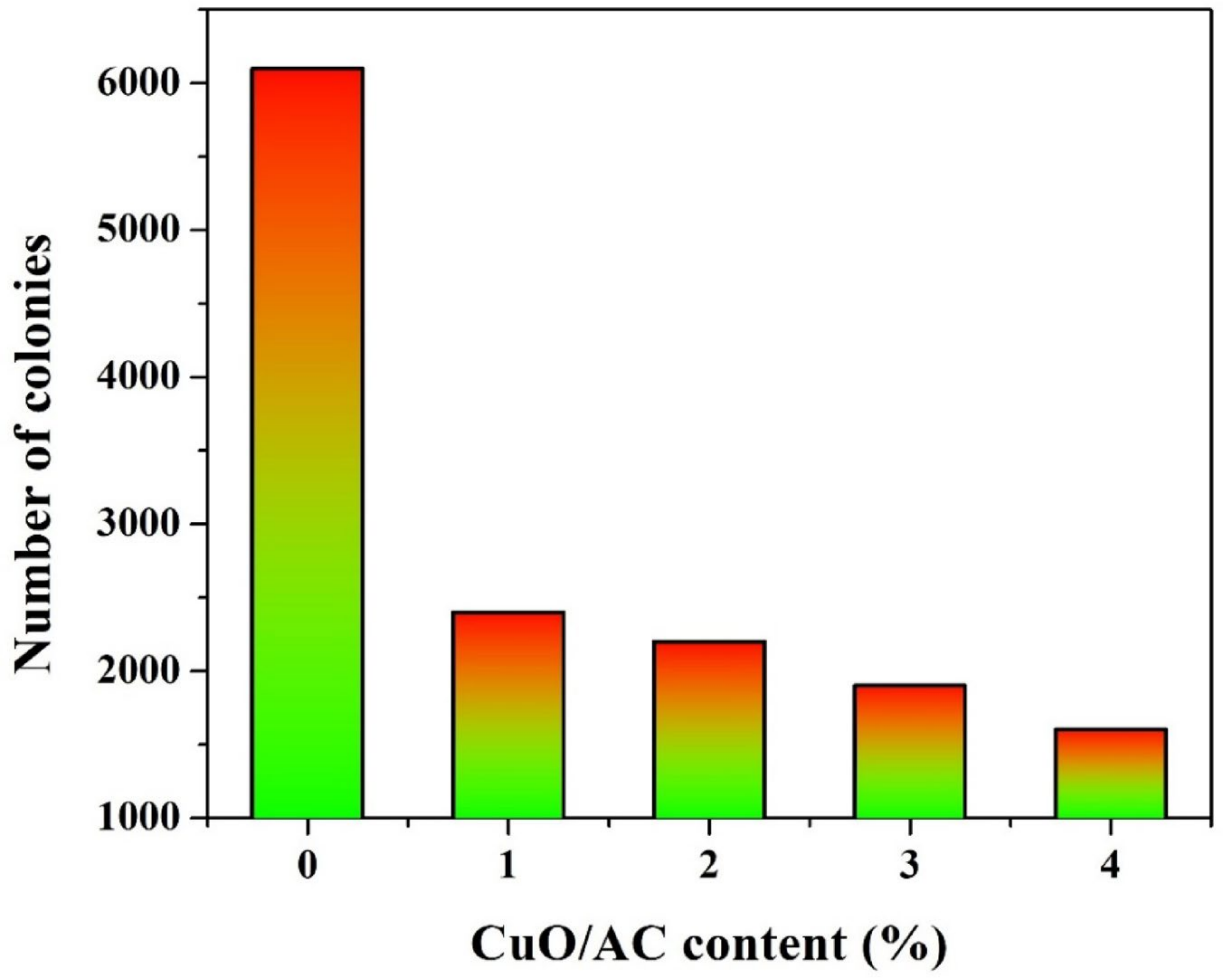
Antibacterial activity of CuO/AC composite showing the reduction in microbial colony count with increasing CuO/AC content in the fabricated cathode.

For antibacterial activity for the proposed cathode, as shown in Fig. 5, the control sample exhibited the highest colony count, exceeding 6000 CFUs, indicating the presence of a dense aerobic bacterial population in the untreated wastewater. However, as CuO/AC content increased, there was a drastic reduction in colony numbers, with 4 wt% CuO/AC reducing the bacterial count to below 1500 CFUs. Even the lowest doping level (1 wt%) exhibited a significant bactericidal effect, cutting the microbial load by more than 60%.

This strong antibacterial activity is primarily attributed to the CuO nanoparticles dispersed on the AC surface. Copper oxide is known to disrupt bacterial cell membranes through oxidative stress and ion leaching, generating reactive oxygen species (ROS) and releasing Cu^2+^ ions that interfere with essential cellular processes such as DNA replication and protein synthesis^30,31^. The high surface area of the activated carbon further enhances the contact between CuO particles and bacterial cells, boosting the material’s antimicrobial efficacy.

From an application perspective, this result is critical. In membrane-less microbial fuel cells, aerobic microorganisms from the wastewater tend to colonize the cathode surface, forming thick biofilms that block oxygen access and interrupt electron transfer, thereby causing a functional disconnection of the cell. This phenomenon, known as cathode fouling, severely compromises the long-term power output and operational stability of MFCs. The introduction of CuO/AC as a cathode catalyst serves a dual role—facilitating oxygen reduction reaction while simultaneously preventing microbial overgrowth.

The observed antibacterial performance confirms that the doped AC composite can effectively suppress aerobic biofilm formation on the cathode, making it particularly suitable for long-term operation of membrane-less MFCs. This advantage is especially pronounced in continuous flow systems, where the fluid movement may distribute bacteria across the cathode, further highlighting the importance of embedding antimicrobial properties directly into the electrode material^32^.

### Electrochemical performance

#### *Effect of CuO content on power generation*

**Fig. 6:**
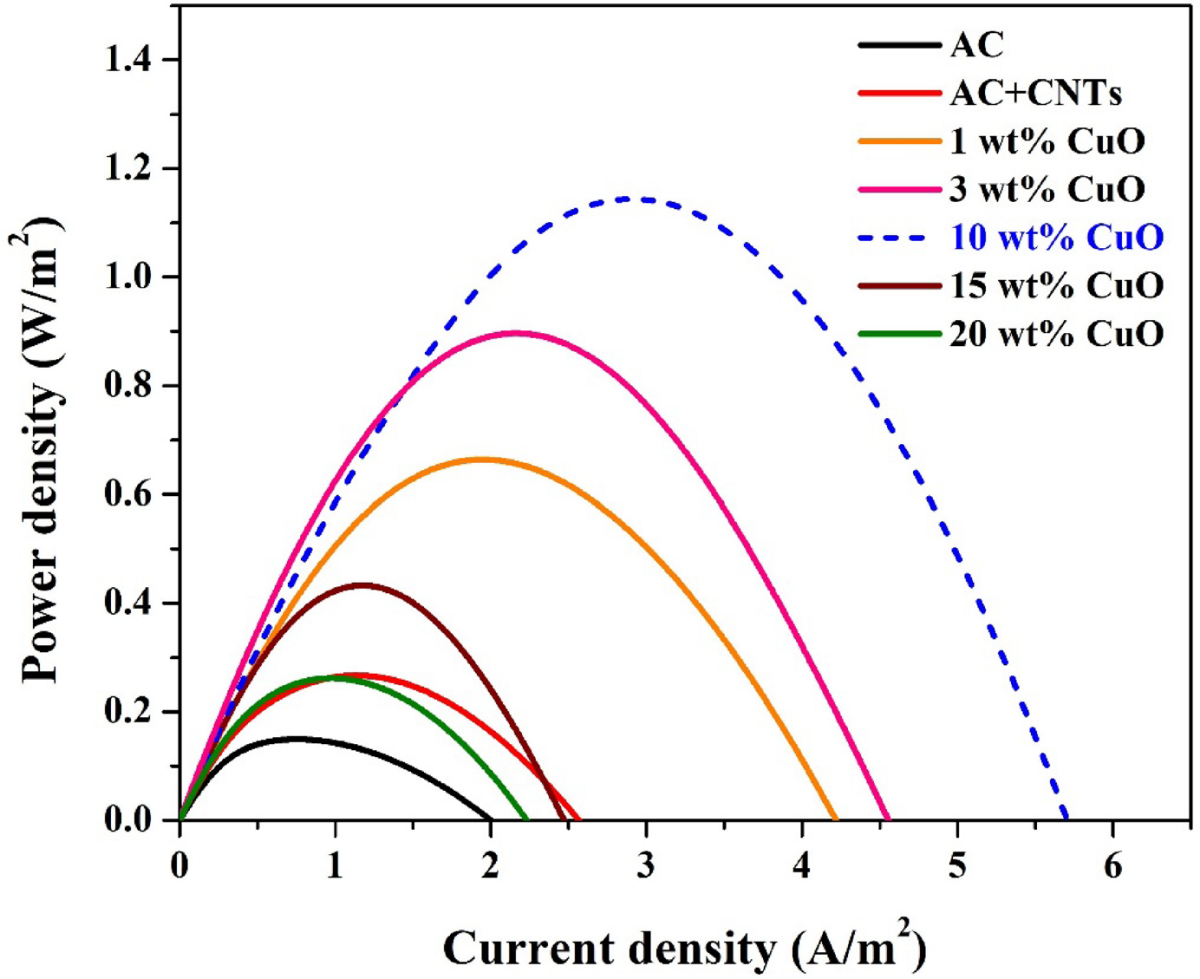
Polarization power density curves of MFCs equipped with cathodes containing varying CuO/AC contents.

To investigate the influence of CuO content on the electrochemical performance of the synthesized cathodes, a series of single-electrode MFCs (each with one graphitized corncob anode and one cathode) were assembled and tested using identical wastewater conditions. The power density versus current density curves (polarization curves) for the different cathode configurations are presented in Fig. 6. The tested cathodes included: pristine activated carbon, AC with carbon nanotubes (AC + CNTs), and AC doped with CuO at 1 wt%, 3 wt%, 10 wt%, 15 wt%, and 20 wt%, each also blended with CNTs to improve conductivity and ORR activity.

**Fig. 7:**
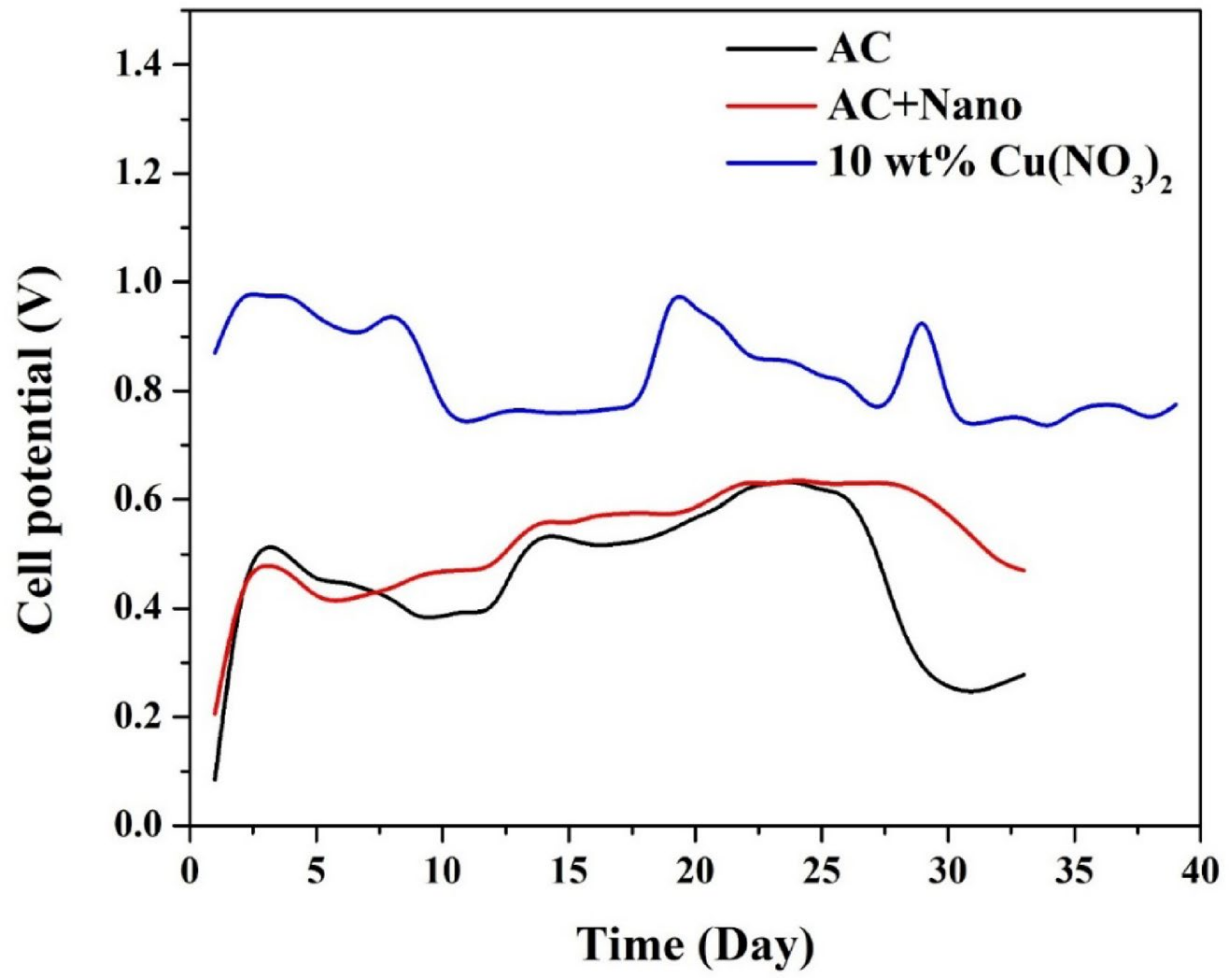
Variation of open cell potential (OCP) over around 40 days for MFCs equipped with AC, AC + CNTs, and 10 wt% CuO-CNT/AC cathodes.

As seen in Fig. 7, the MFC equipped with the 10 wt% CuO-CNT/AC cathode exhibited the highest electrochemical performance, achieving a maximum power density of approximately 1.25 W/m^2^ and a corresponding maximum current density of around 5.2 A/m^2^. This indicates a significant improvement compared to the pristine AC cathode, which showed a peak power density below 0.25 W/m^2^ and current density under 1.2 A/m^2^.

The improved performance of the 10 wt% CuO cathode can be attributed to the optimal balance between CuO content and carbon conductivity^33^. At this loading, CuO nanoparticles are well-dispersed across the carbon matrix (as confirmed by SEM and elemental mapping), providing abundant catalytic sites for the oxygen reduction reaction. Moreover, the inclusion of CNTs enhances electrical conductivity and promotes efficient electron transport across the electrode surface.

At lower CuO contents (1 wt% and 3 wt%), the enhancement in power density is evident but not optimal. This is likely due to insufficient CuO active sites, which limit the ORR kinetics and reduce cathodic efficiency. Conversely, at higher CuO contents (15 wt% and 20 wt%), a decline in performance is observed. This can be explained by excessive CuO loading, which may lead to particle agglomeration and blockage of the carbon surface pores, thereby reducing the effective surface area and impeding oxygen diffusion.

Additionally, high CuO loading may introduce excess internal resistance or disrupt the conductive network within the cathode due to CuO’s relatively low conductivity compared to carbon-based materials. These negative effects outweigh the catalytic benefits at higher dopant levels, resulting in reduced power output.

The data collectively demonstrate that a CuO content of 10 wt% offers the best performance, indicating that this composition achieves an optimal compromise between catalytic activity, conductivity, and structural integrity. This cathode formulation is especially promising for scale-up in membrane-less MFCs, where fouling resistance and ORR efficiency are both critical for sustained performance.

#### *Long-Term stability: open cell potential monitoring*

To assess the long-term stability and electrochemical sustainability of the synthesized cathodes, the open cell potential (OCP) of the microbial fuel cells was monitored continuously over a period of 37 days. The results are presented in Fig. 7, comparing three cathode configurations: Pristine AC, AC + CNTs and CuO-doped AC (10 wt% Cu(NO₃)₂) with CNTs.

As illustrated in Fig. 7, the 10 wt% CuO-CNT/AC-based MFC exhibited the highest and most stable OCP throughout the operational period. The cell potential quickly rose within the first 2–3 days and stabilized at a value around 0.85–0.95 V, peaking at approximately 1.05 V. This performance remained consistently superior to the other two configurations, indicating excellent electrocatalytic activity and resistance to cathode fouling.

In contrast, the AC-based MFC reached a maximum OCP of ~ 0.6 V around day 5, but the voltage gradually declined after day 25, eventually dropping below 0.4 V by day 35. This decline is commonly associated with biofouling and oxygen mass transfer limitations on the cathode surface, which is expected for non-modified activated carbon in membrane-less systems where aerobic microorganisms can colonize the electrode.

The MFC using AC + CNTs showed slightly improved performance over pristine AC, reaching a stable OCP of approximately 0.6–0.65 V up to day 30, after which a modest decline was observed. The addition of CNTs enhanced the electrical conductivity and surface reactivity to some extent but was insufficient to prevent long-term biological fouling.

The superior performance of the 10 wt% CuO-based cathode can be attributed to two synergistic effects: Enhanced oxygen reduction reaction kinetics due to the catalytic activity of well-dispersed CuO nanoparticles. Antibacterial properties of CuO, which inhibit biofilm formation and maintain surface activity, as previously confirmed by antibacterial colony reduction tests^34^.

Furthermore, the stability of the OCP curve for the CuO-doped system suggests sustained interaction between the biocatalyst (anode biofilm) and the cathode, with minimal internal resistance buildup. This is especially crucial for real wastewater applications, where long-term operation without membrane protection often leads to rapid cathode degradation in standard materials.

#### *Current density stability during long-term operation*

**Fig. 8:**
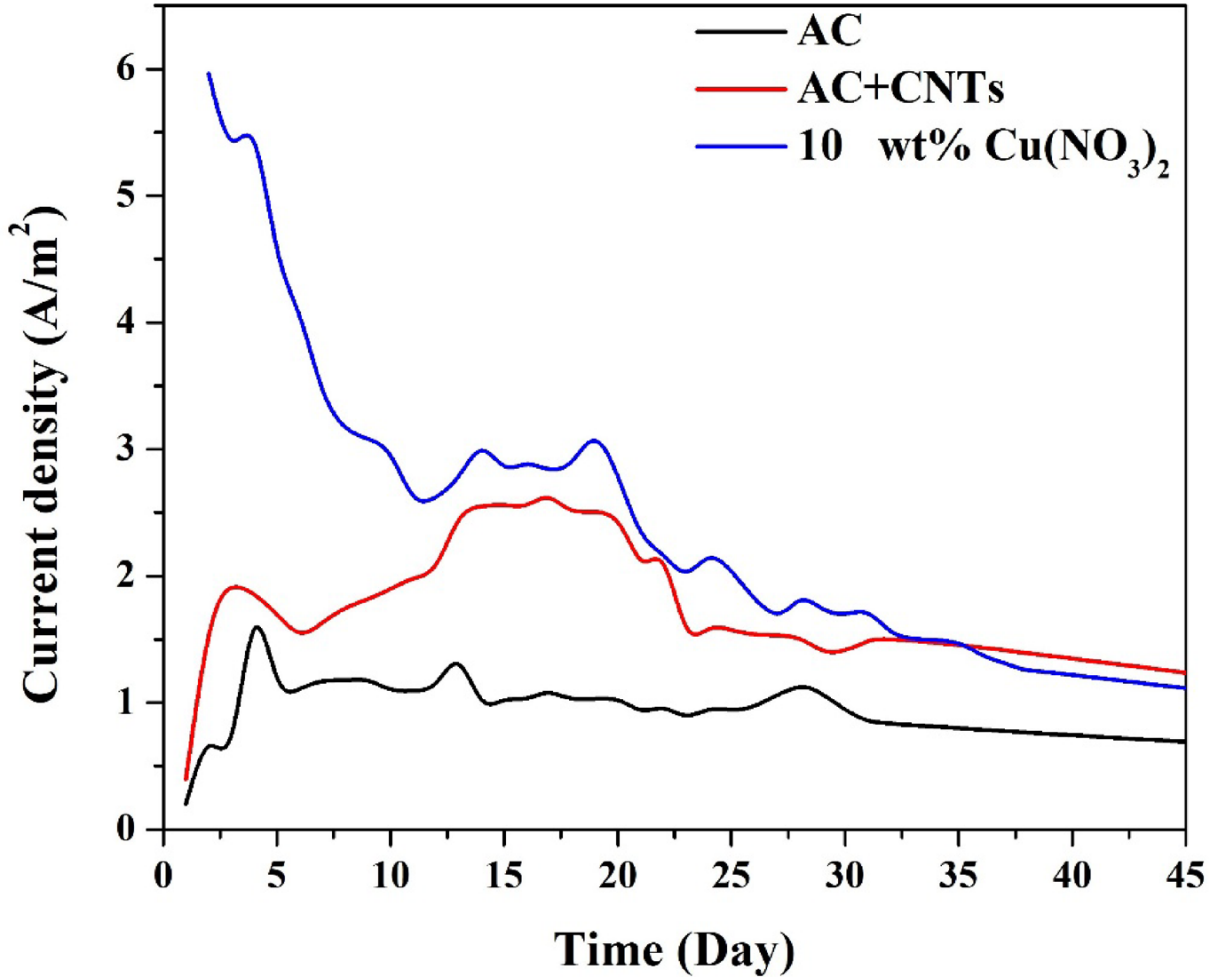
Long-term current density profiles of MFCs with different cathode materials. The 10 wt% CuO-CNT/AC cathode exhibited the highest and most sustained current output.

The current density output of MFCs equipped with different cathode materials was monitored over around 40 days to evaluate the electrochemical durability and sustainability of each configuration under continuous operation with real wastewater. The results are presented in Fig. 8, showing the performance of pristine activated carbon, AC with carbon nanotubes, and the optimized 10 wt% CuO-doped AC with CNTs.

As shown in Fig. 8, the MFC with the 10 wt% CuO-CNT/AC cathode exhibited the highest initial current density, peaking at nearly 6 A/m^2^ within the first two days. This impressive performance can be attributed to the enhanced electrocatalytic activity of CuO for oxygen reduction and the high conductivity of the CNT network facilitating electron transfer.

After the initial peak, a gradual decline was observed—typical in long-term MFC operation due to biofilm dynamics, electrode aging, or nutrient depletion—but the current density remained above 2 A/m^2^ for over 25 days, demonstrating excellent long-term performance and resistance to cathode fouling. Even toward the end of the monitoring period, the current density stabilized near 1.3 A/m^2^, remaining higher than both the AC and AC + CNTs systems.

The MFC using AC + CNTs reached a maximum current density of approximately 2.5 A/m^2^, with a more gradual decline and final stabilization near 1.2 A/m^2^, showing improved conductivity but lacking the antibacterial properties of CuO to prevent biofilm accumulation.

In contrast, the AC-only cathode exhibited the poorest performance, peaking at just over 1.5 A/m^2^ and stabilizing at ~ 0.9 A/m^2^. This reflects the limited ORR activity and the susceptibility of the cathode surface to biofouling, which compromises oxygen accessibility and electron flow.

While both cathodes exhibited comparable current densities after approximately 35 days of operation, the CuO/AC-based cathode demonstrated superior performance during the initial and mid-term phases, particularly in terms of peak current density, COD removal efficiency, and resistance to microbial contamination. This enhanced behavior is attributed to the electrocatalytic nature and antibacterial activity of the CuO particles. Although a minor decline in current density was observed after extended operation, this does not overshadow the improved early-stage performance and overall operational stability. Therefore, the CuO-containing cathode presents a compelling candidate for applications requiring high initial output and reduced biofouling over moderate durations.

Although biogas formation was not directly monitored, the use of air-cathodes, short retention times, and the antimicrobial nature of CuO likely suppressed methanogenic activity, as methanogens require strictly anaerobic conditions and are sensitive to metal oxide toxicity.

#### *Power density evaluation during long-term operation*

**Fig. 9:**
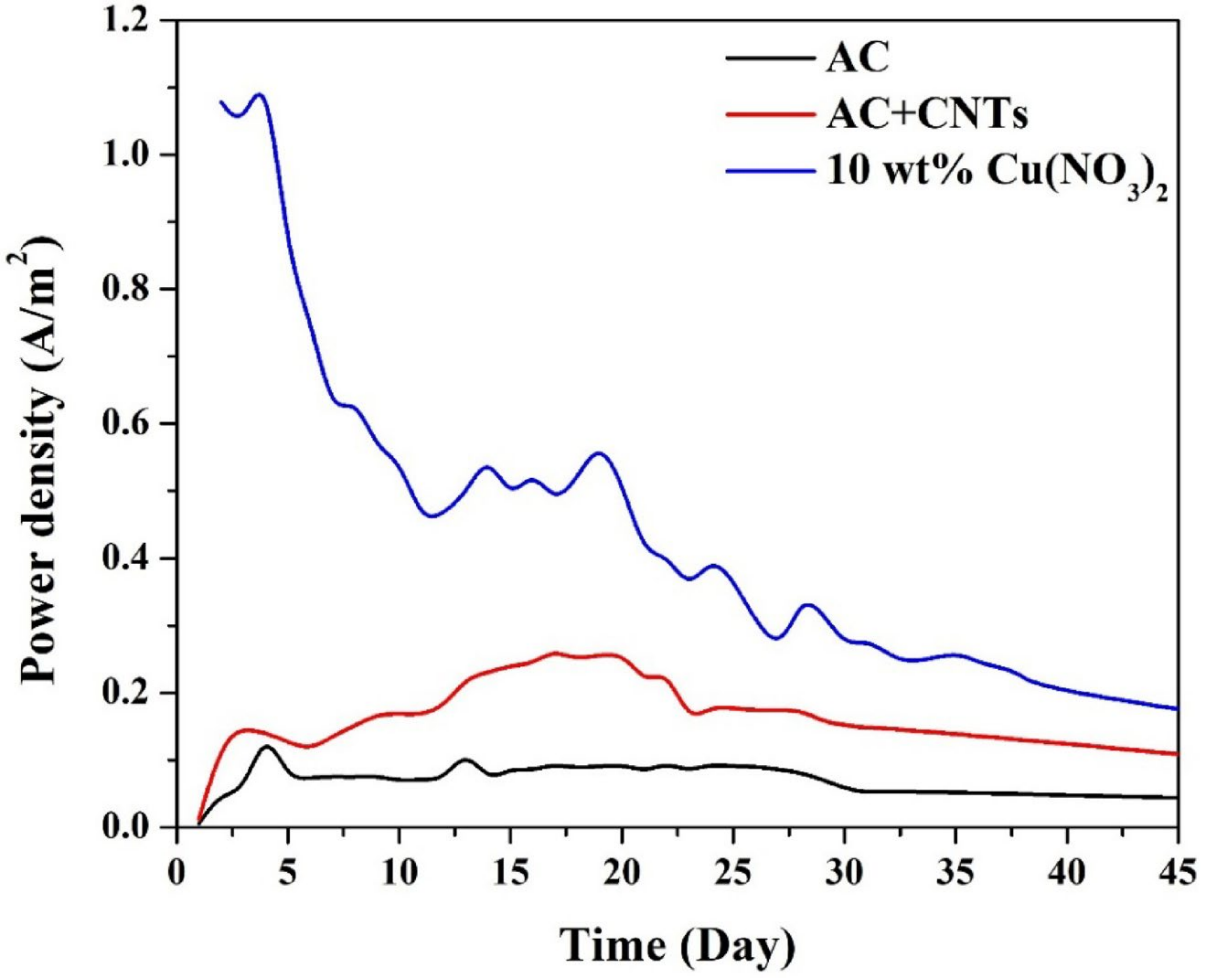
Long-term power density output of MFCs using different cathodes. The 10 wt% CuO-CNT/AC cathode delivered the highest and most stable power generation.

To further assess the overall performance of the MFCs, the power density output was tracked under identical operating conditions using three different cathode configurations. The results are shown in Fig. 9. The MFC with the 10 wt% CuO-CNTs/AC cathode exhibited a significantly higher power density than the other two systems throughout the operational period. It reached an initial peak of approximately 1.1 W/m^2^ in the first few days, then gradually declined due to factors such as substrate depletion, biofilm evolution, and potential cathode surface changes. Despite this decline, the CuO-based MFC maintained a power output above 0.3 W/m^2^ for over 30 days, indicating excellent long-term performance and electrode durability.

In contrast, the AC + CNTs cathode showed improved performance over pristine AC due to better conductivity and enhanced surface area from the nanotubes. It stabilized around 0.25 W/m^2^ and maintained a relatively steady output for most of the operation, but lacked the biocidal capability of CuO to prevent microbial colonization, which can contribute to a performance plateau.

The pristine AC cathode, lacking both enhanced conductivity and antimicrobial features, showed the lowest power output, peaking at only ~ 0.15 W/m^2^ and stabilizing below 0.1 W/m^2^. This poor performance reflects the typical issues in membrane-less MFCs, such as cathode fouling by aerobic bacteria, limited ORR efficiency, and insufficient electrode conductivity.

The data reinforce the dual functionality of the CuO/CNTs-enhanced cathode: it promotes efficient oxygen reduction reaction and inhibits biofouling, both of which are essential for sustained power generation in membrane-less MFCs operating with complex real-world wastewater.

#### *Surface morphological analysis of used cathodes (optical microscopy)*

To provide further insight into the performance enhancement mechanisms of the CuO-containing cathode in MFC operation, high-resolution optical microscopy was used to examine the post-operation surface of cathodes after 43 days of batch-mode operation. The cathodes analyzed were: the pristine AC-CNTs cathode, and the CuO-doped AC (10 wt%) cathode. Each cathode was imaged at two magnifications: 200 μm and 100 μm.

**Fig. 10:**
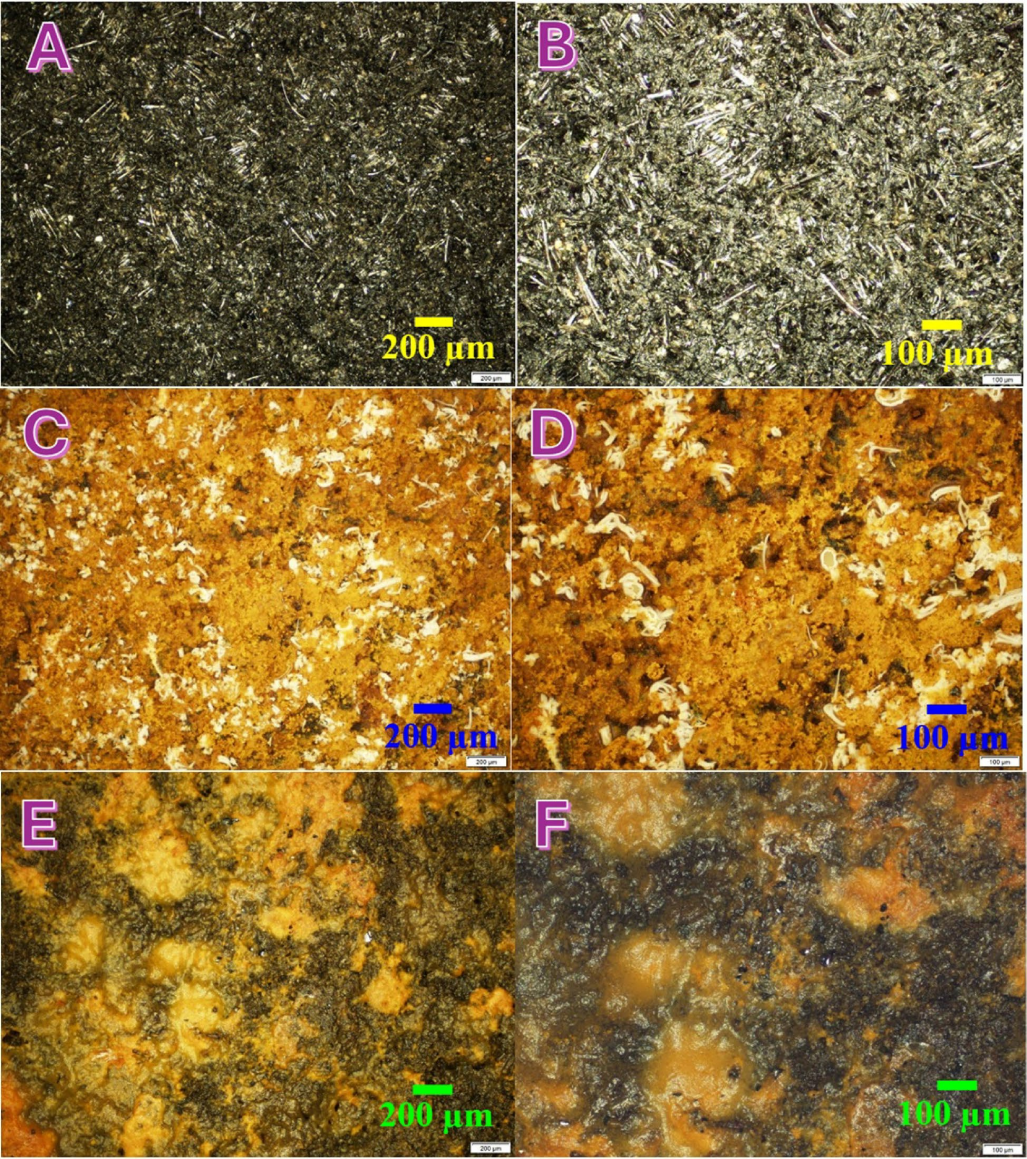
High-resolution Optical microscopy images of MFC cathodes. (**A**, **B**) Unused cathode; (**C**, **D**) used AC-CNTs cathode showing heavy biofilm and microbial colonies; (**E**, **F**) used 10 wt% CuO/AC cathode showing clean surface with minimal fouling, confirming the antibacterial effect of CuO.

The first set of images (Fig. 10A and B) represents the surface of the unused cathodes before MFC operation. Both the AC-CNT and CuO/AC cathodes displayed similarly clean and uniform textures with visible fibrous or granular morphology from the conductive carbon materials and embedded nanostructures. No contamination, fouling, or deposits were visible, which provides a reference for evaluating surface changes due to biofouling or operation-related degradation.

The second set of images (Fig. 10C and D), taken from the used AC-CNTs cathode, clearly exhibits significant biofouling and surface coverage. Numerous white, filamentous or colony-like structures are dispersed throughout the surface, which are consistent in appearance with microbial colonies—a typical indicator of aerobic bacterial growth in membrane-less MFCs. Furthermore, a dense, sponge-like layer composed of fine agglomerated particles can be seen overlaying the surface. This is most likely a biofilm matrix formed by microbial secretions and accumulated organic matter.

The development of this continuous biofilm layer impedes oxygen diffusion to the cathode and physically insulates the active sites from participating in the ORR, which explains the observed decline in cell potential, current density, and power output in the AC-CNTs MFC. This biofouling-induced deterioration is a well-known issue in membrane-less MFCs when no antibacterial mechanism is present.

In contrast, the third set of images, corresponding to the used CuO-doped AC cathode (Fig. 10E and F), reveals a distinctly different surface morphology. Most notably, no microbial colonies or white biofilm patches are observed. The surface remains relatively clear and intact, with visible bare black regions and isolated, non-uniform deposits. These deposits appear as randomly distributed, amorphous regions that are likely attributable to adsorbed organic/inorganic waste residues, rather than biological growth.

The absence of bacterial growth and dense biofilm formation confirms the antibacterial effectiveness of CuO. CuO nanoparticles are known to exert antimicrobial activity through mechanisms such as: Generation of reactive oxygen species (ROS), release of Cu^2+^ ions that disrupt cellular membranes, and interference with microbial respiration and DNA replication.

This antibiofouling behavior is directly responsible for maintaining cathodic activity and ORR kinetics over extended operation. The ability of CuO to preserve the active surface, combined with enhanced ORR catalytic properties, explains the superior and more stable performance of the CuO/AC cathode observed in the electrochemical tests (higher OCP, power, and current densities).

Overall, the microscopy analysis provides compelling visual evidence that CuO incorporation suppresses cathode biofouling and helps maintain long-term functionality in membrane-less MFCs. In contrast, AC-CNTs cathodes, despite improved conductivity, suffer from microbial colonization that degrades electrochemical performance. These results validate the dual-functional role of CuO as both an ORR catalyst and antibacterial agent, supporting its application in practical, low-maintenance bio electrochemical systems.

### 3.3. Influence of operation mode

#### *Open cell potential*

**Fig. 11:**
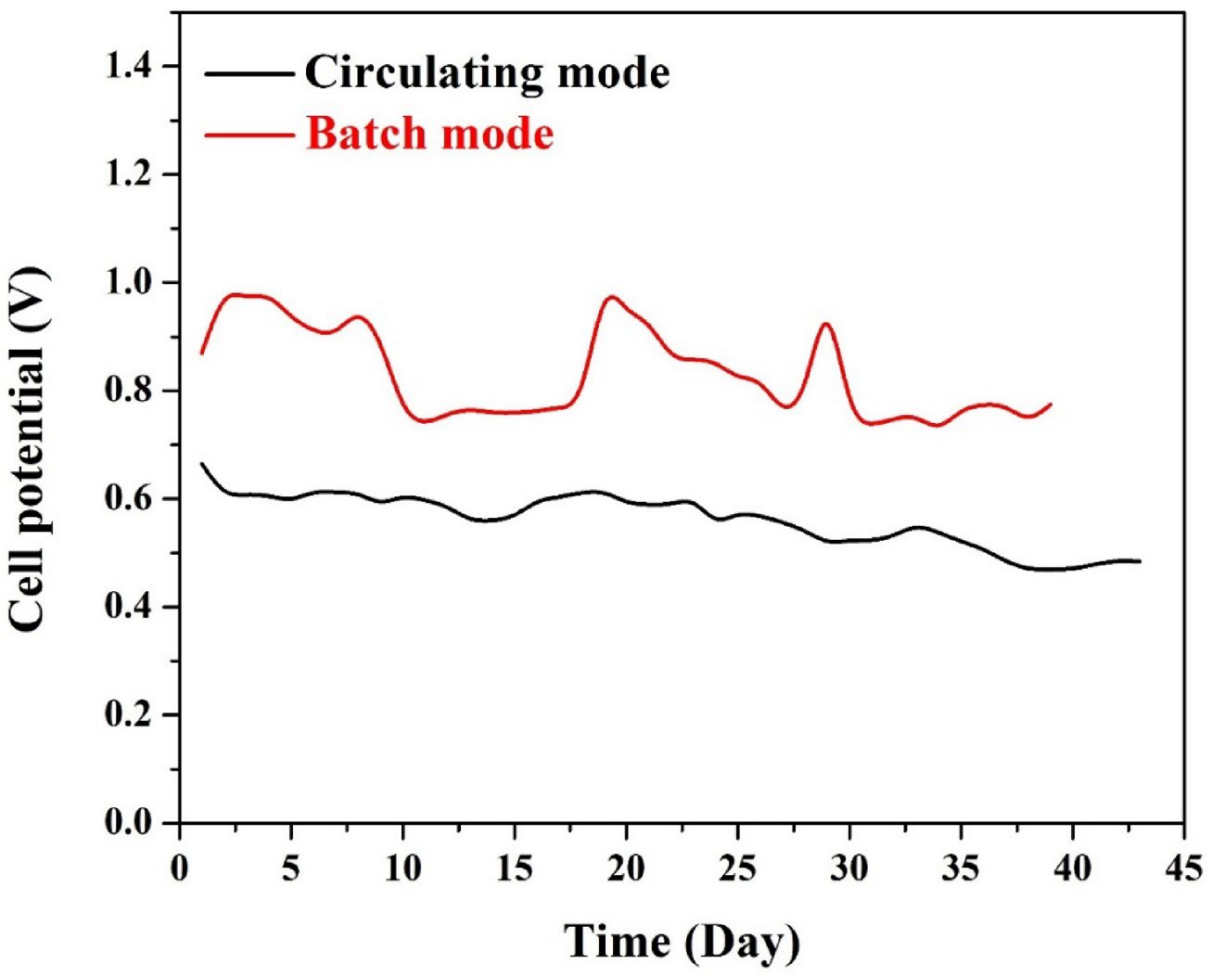
Comparison of open cell potential (OCP) for MFCs operated in batch and continuous (circulating) modes using 10 wt% CuO-CNT/AC cathode.

To investigate the impact of operation mode on MFC performance, two identical systems were assembled using the optimized 10 wt% CuO-CNT/AC cathode, with one operated in batch mode and the other in continuous circulation mode. The OCP of both systems was monitored for around 40 days, and the results are illustrated in Fig. 11.

The batch-mode MFC consistently exhibited a higher OCP, fluctuating between ~ 0.85–1.0 V during the entire operation. This higher potential can be attributed to more stable substrate concentration and higher retention time, allowing electroactive microorganisms at the anode to fully oxidize organic matter and maintain a high electron generation rate^25,35^. Additionally, the stationary condition may result in a relatively higher dissolved oxygen concentration at the cathode surface during periods of limited bacterial colonization, thus enhancing the cathodic ORR.

In contrast, the circulating (continuous) mode MFC maintained a lower but more stable OCP, typically ranging between 0.58 and 0.68 V. While the peak voltages are lower compared to batch mode, the stability of the signal over time reflects a more uniform and consistent electrochemical environment. The continuous supply and removal of wastewater in this mode prevent the accumulation of toxic byproducts or substrate depletion, leading to enhanced operational resilience.

Several factors contribute to the observed difference:

- Lower OCP in continuous mode may result from dilution effects or transient substrate concentration at the anode, as the feed is constantly refreshed^36^.
- However, the enhanced fluid dynamics improve oxygen diffusion to the cathode and reduce the accumulation of aerobic microbial biofilms, especially in membrane-less designs.
- The mechanical shear stress caused by circulating liquid can help suppress biofouling at the cathode surface, promoting longer-term performance even if initial voltage is lower^37^.
- In batch mode, although initial voltages are higher, the performance shows more fluctuations, which may reflect intermittent changes in microbial activity, substrate consumption, or local pH/electrolyte conditions due to stagnant flow.

From a practical standpoint, the more stable performance of the continuous mode is highly advantageous for industrial applications, where predictability, robustness, and integration into ongoing waste streams are more critical than occasional high output values.

The internal resistance of the MFC was estimated from the slope of the linear region in the polarization curve derived from linear sweep voltammetry data. For the CuO/AC-based MFC operating under continuous mode, the internal resistance was approximated to be 71.5 Ω. Although individual components—activation, ohmic, and concentration overpotentials—could not be separately quantified, qualitative analysis of the polarization curve indicated distinct regions corresponding to each: an initial voltage drop at low current (activation losses), a linear mid-region (ohmic resistance), and a plateau at high current (concentration overpotentials). These observations align with the standard performance characteristics of membrane-less MFCs.

#### *Current density*

**Fig. 12:**
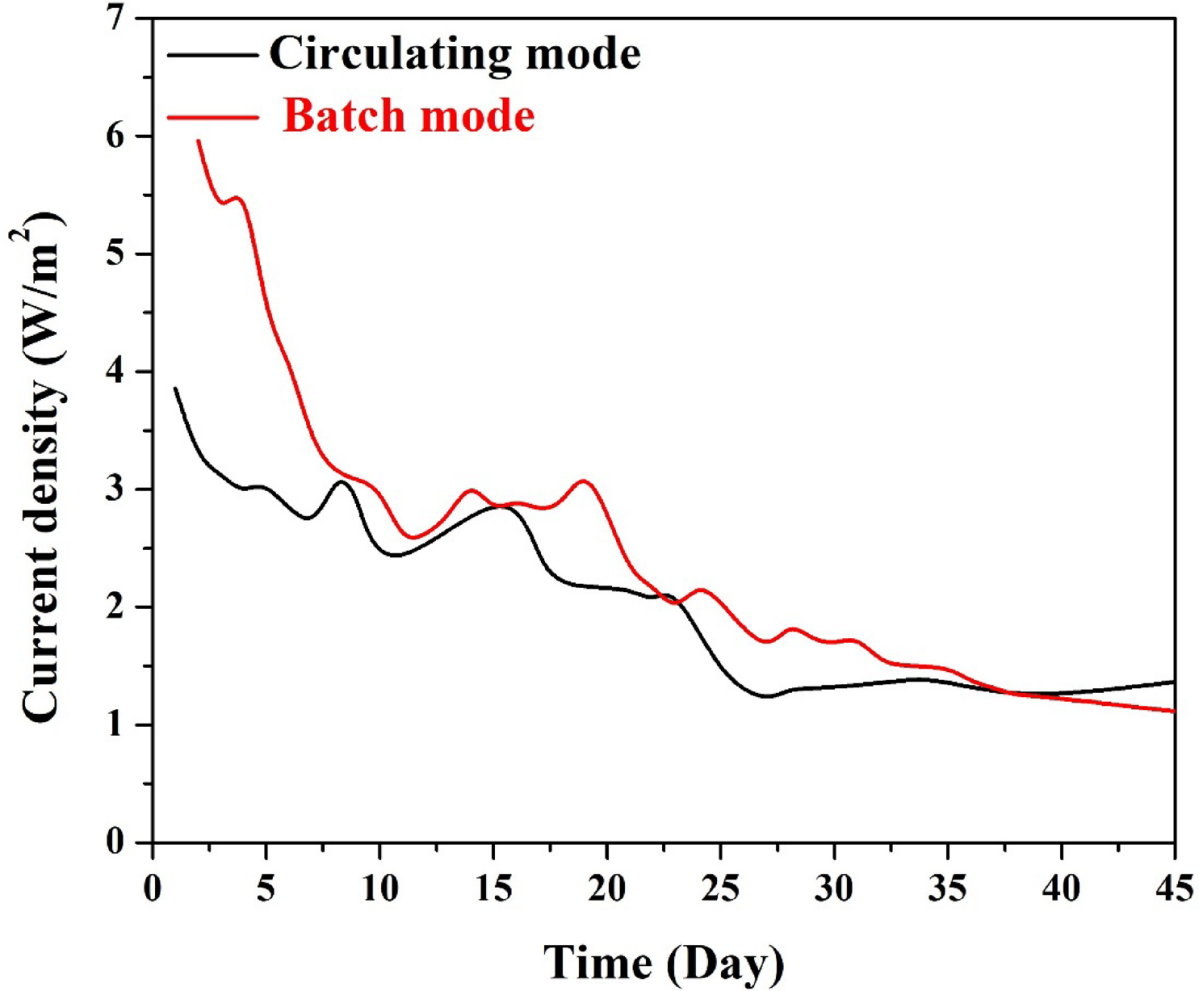
Current density profiles of MFCs operated in batch and continuous modes using 10 wt% CuO-CNT/AC cathode.

To evaluate the effect of operation mode on electrical output, the current density profiles of the MFCs operated in batch mode and circulating (continuous) mode were monitored for 43 days using the optimized 10 wt% CuO-CNT/AC cathode. The results are depicted in Fig. 12.

In the early phase (days 1–10), the batch-mode MFC demonstrated significantly higher current densities, peaking above 6 A/m^2^, due to the initial abundance of organic substrates and the well-developed electroactive biofilm under stagnant conditions. However, this high performance began to decline sharply, dropping to below 2 A/m^2^ after 25 days, likely due to substrate depletion, localized pH shifts, and especially biofilm overgrowth on the cathode surface that impaired oxygen transfer and electrode function.

Conversely, the continuous mode MFC showed a more moderate but stable trend, with an early peak around 3.7 A/m^2^, followed by a slower rate of decline. It maintained current densities between 2.0 and 2.5 A/m^2^ for more than 20 days, and unlike the batch mode, showed signs of recovery and stabilization toward the end of the monitoring period. This reflects the benefits of constant nutrient replenishment, better mass transfer, and reduced accumulation of inhibitory byproducts in the continuous system.

Although batch mode initially outperformed the circulating mode, the latter showed more stable long-term behavior, with fewer sharp fluctuations and better maintenance of electrochemical function. The improved cathodic performance over time in the circulating mode can also be attributed to reduced aerobic fouling at the cathode due to continuous shear forces and regular renewal of electrolyte near the electrode surface. Overall, this comparison highlights that while batch mode may be beneficial for short-term peak output, the continuous mode is more suitable for stable, scalable, and reliable long-term operation, especially when using a membrane-less MFC configuration.

#### *Power density*

**Fig. 13:**
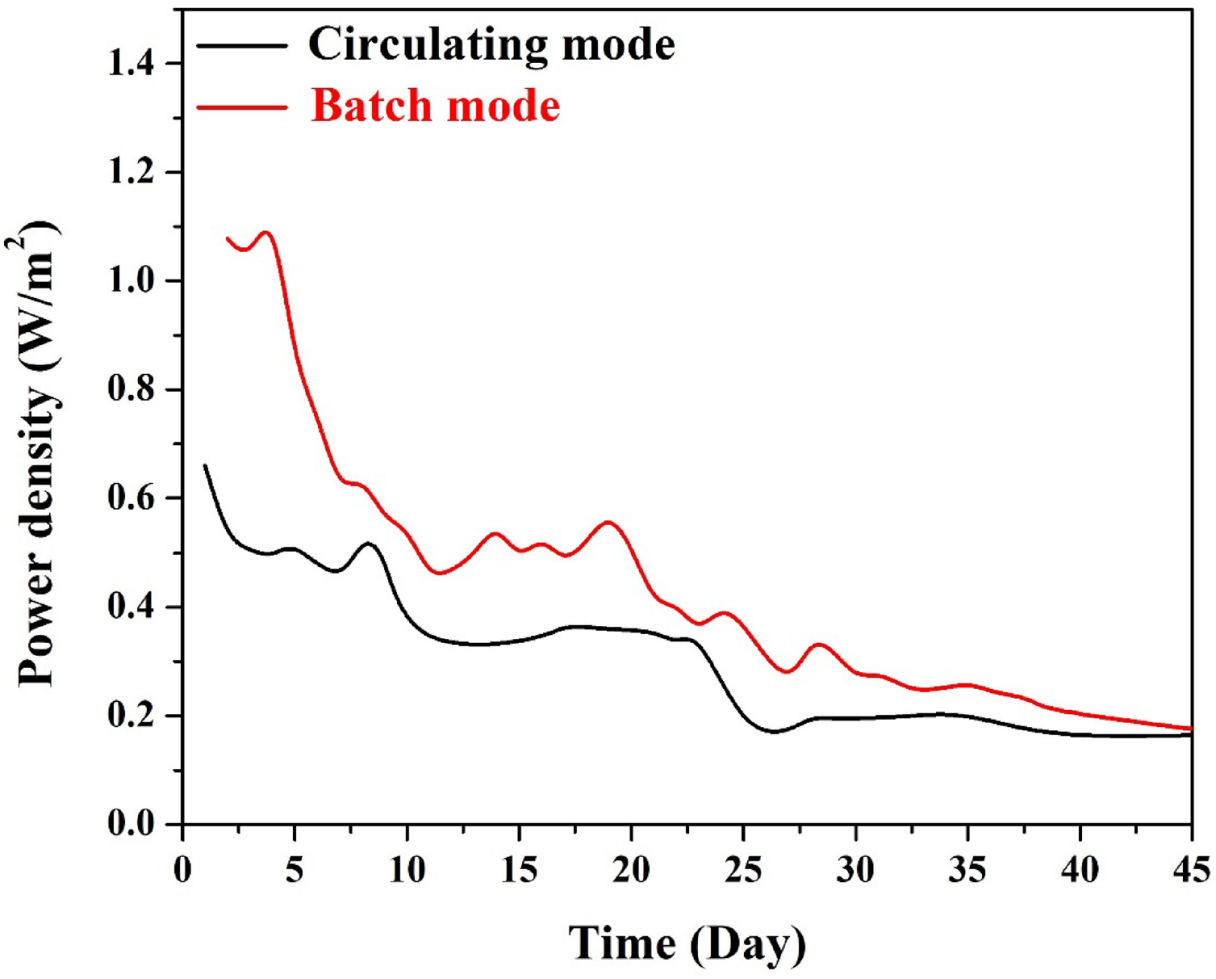
Power density output of MFCs operated in batch and circulating modes using 10 wt% CuO-CNT/AC cathode.

The power density output of microbial fuel cells operated in batch and circulating modes was monitored, and the results are presented in Fig. 13. In the initial days of operation, the batch mode MFC exhibited a significantly higher power density, peaking at around 6 W/m^2^, owing to the high substrate availability and stable anode-biofilm interaction in the absence of flow. However, this performance declined steadily over time, falling below 2 W/m^2^ by day 25, and continued to drop gradually due to substrate depletion, localized environmental fluctuations, and most importantly, biofilm formation on the cathode surface—a common issue in stagnant, membrane-less systems.

In contrast, the circulating mode showed a lower initial peak (approximately 3.8 W/m^2^), yet demonstrated superior stability over time. Between days 5 and 25, the system maintained a relatively steady output between 2.5 and 3.0 W/m^2^, with less fluctuation and a slower rate of decline. This stability can be attributed to the constant flow of wastewater, which continuously replenishes nutrients, removes inhibitory byproducts, and helps mitigate cathode fouling through hydrodynamic shear.

After about 30 days, both systems converged to similar power levels near 1 W/m^2^, but it is noteworthy that the circulating system exhibited a slight recovery and stabilization toward the end, unlike the batch system, which showed a continued gradual decline. The results clearly highlight the trade-off between short-term peak performance and long-term operational stability. While batch mode offers higher initial power output, it is more vulnerable to environmental imbalances and fouling. The continuous mode, though yielding lower peak values, offers a more consistent and robust performance, which is more desirable in industrial and long-term deployment scenarios.

#### *COD removal efficiency*

**Fig. 14:**
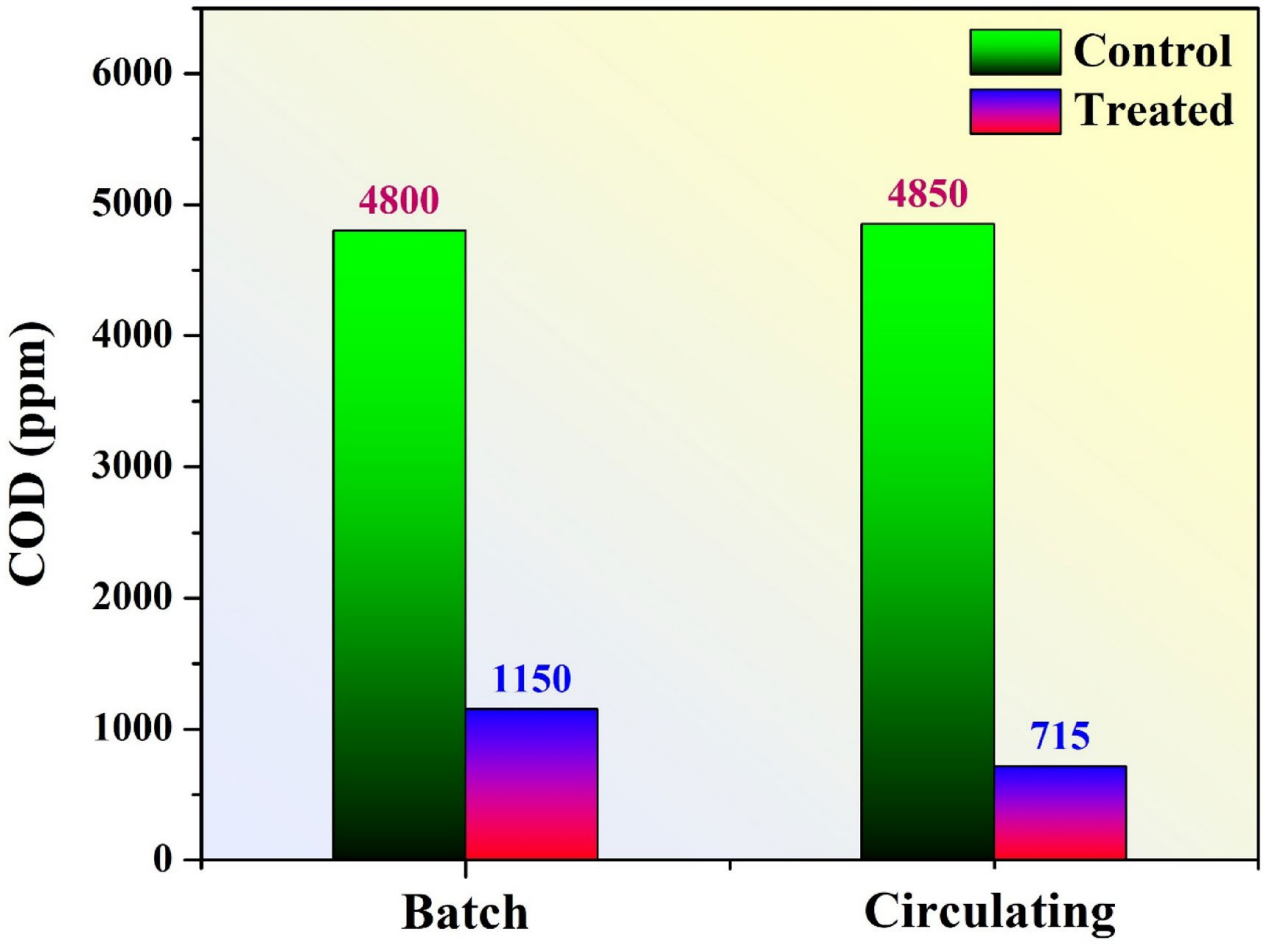
COD levels of wastewater before (green bars) and after treatment (blue bars) in batch and circulating mode MFCs.

The COD is a key indicator of organic pollutant concentration in wastewater. In this study, COD measurements were taken before and after treatment using MFCs operated in both batch and circulating modes to assess the effectiveness of organic matter degradation. The results are illustrated in Fig. 14.

The initial COD values of the real industrial wastewater used in both systems were approximately 4800 ppm for the batch mode and 4850 ppm for the circulating mode, confirming the comparability of the starting conditions. After treatment, the COD value dropped to 1150 ppm in the batch system and further down to 715 ppm in the circulating mode, indicating significant removal of organic content in both cases. Consequently, the estimated COD removal is around 76% and 85.3% for the batch and continuous cells, respectively.

These results clearly demonstrate that while both configurations are effective in treating high-strength organic wastewater, the circulating mode outperforms batch mode in terms of degradation efficiency. This can be attributed to the continuous replenishment of substrate, improved contact between electroactive microorganisms and organics, and better oxygen availability at the cathode in the circulating system. Additionally, the constant flow helps prevent the accumulation of metabolic byproducts and supports a more stable microbial community, which in turn improves long-term degradation rates.

Therefore, the integration of continuous flow into MFC operation not only enhances electrical performance stability, as previously shown, but also improves wastewater treatment efficiency, making it a more viable configuration for real-world applications where high throughput and consistent treatment quality are required.

## Conclusion

Copper oxide nanoparticles-incorporated activated carbon can be synthesized, with different metal oxides, by impregnation of the activated carbon in copper nitrate/ethanol solution followed by drying and thermal treatment. The produced composite possesses distinct antibacterial and electrocatalytic activity toward oxygen reduction reaction depending on the metal oxide content. The findings revealed that CuO incorporation not only improved the oxygen reduction reaction kinetics but also provided a potent antibacterial effect, which played a critical role in mitigating cathode biofouling—a major challenge in membrane-less configurations. This was clearly supported by long-term operation and post-experiment surface analyses, where the CuO-containing cathode maintained its integrity and exhibited minimal microbial colonization, while the AC-CNTs cathode was heavily fouled. Furthermore, the study emphasized the significance of operation mode on system performance, with continuous circulation proving advantageous in terms of stability and COD removal, despite exhibiting slightly lower initial electrochemical outputs compared to batch mode. These outcomes collectively underline the potential of CuO/AC cathodes in extending the lifespan and reliability of membrane-less MFC systems, and demonstrate the industrial relevance of adopting continuous operational strategies for sustainable wastewater treatment coupled with energy recovery.

## Data Availability

All data generated or analyzed during this study are included in this published article.
